# A Circulating Subpopulation of Monocytic Myeloid-Derived Suppressor Cells as an Independent Prognostic/Predictive Factor in Untreated Non-Small Lung Cancer Patients

**DOI:** 10.1155/2014/659294

**Published:** 2014-11-11

**Authors:** Eleni-Kyriaki Vetsika, Filippos Koinis, Marianthi Gioulbasani, Despoina Aggouraki, Anna Koutoulaki, Eirini Skalidaki, Dimitris Mavroudis, Vassilis Georgoulias, Athanasios Kotsakis

**Affiliations:** ^1^Laboratory of Cancer Cell Biology, School of Medicine, University of Crete, 71110 Heraklion, Crete, Greece; ^2^Department of Medical Oncology, University Hospital of Heraklion, 71110 Heraklion, Crete, Greece

## Abstract

Myeloid-derived suppressor cells (MDSCs) represent a heterogeneous population of cells with immunosuppressive properties and might confer to worse prognosis in cancer patients. The presence of phenotypically newly described subpopulations of MDSCs and their association with the clinical outcome were investigated in non-small cell lung cancer (NSCLC) patients. The percentages and correlation between MDSCs and distinct immune cells in the peripheral blood of 110 chemotherapy-naive patients before treatment and healthy controls were investigated using flow cytometry. Two monocytic [CD14^+^CD15^−^CD11b^+^CD33^+^HLA-DR^−^Lin^−^ and CD14^+^CD15^+^CD11b^+^CD33^+^HLA-DR^−^Lin^−^] and a granulocytic [CD14^−^CD15^+^CD11b^+^CD33^+^HLA-DR^−^Lin^−^] subpopulations of MDSCs were identified, expressing inducible nitric oxide synthase, and reactive oxygen species, respectively. Increased percentages of both monocytic-MDSCs' subpopulations were inversely correlated to dendritic/monocyte levels (*P* ≤ 0.04), while granulocytic-MDSCs were inversely correlated to CD4^+^ T cells (*P* = 0.006). Increased percentages of monocytic-MDSCs were associated with worse response to treatment (*P* = 0.02) and patients with normal levels of CD14^+^CD15^+^CD11b^+^CD33^+^HLA-DR^−^Lin^−^ had longer overall survival and progression free-survival compared to those with high levels (*P* = 0.008 and *P* = 0.005, resp.). Multivariate analysis revealed that the increased percentages of CD14^+^CD15^+^CD11b^+^CD33^+^HLA-DR^−^Lin^−^ MDSCs were independently associated with decreased progression free-survival and overall survival. The data provide evidence that increased percentages of new monocytic-MDSCs' subpopulations in advanced NSCLC patients are associated with an unfavourable clinical outcome.

## 1. Introduction

Lung cancer is the major cause of cancer-related death in many developed countries. Non-small cell lung cancer (NSCLC) is the most common type (about 85%) of lung cancer [1]. However, the overall survival (OS) of the majority of patients with NSCLC receiving conventional cancer treatment such as surgery, radiotherapy, and chemotherapy remains low [2]. Immunotherapy is an attractive therapeutic option that has been increasingly used against several types of cancer targeting antigens derived from cancer cells and enforcing patient's immune system. Nevertheless, most of the clinical studies with cancer immunotherapy, so far, have failed to demonstrate a clear clinical benefit [3].

A possible explanation is that activation of the immune system alone is not capable of inducing a sufficient response to therapy since other mechanisms, such as immune suppression, are involved. Therefore, combined therapies that on one hand induce immune activation and on the other hand inhibit suppressive mechanisms could be considered necessary to develop an effective immunologic strategy against cancer [4].

Myeloid-derived suppressor cells (MDSC) [5], T regulatory cells (Tregs) [6], and T helper 17 (Th17) cells [7] have been characterized as suppressive cells targeting both innate and adaptive immunity. These cells exert their suppressive action through several mechanisms including the release of inhibitory cytokines such as interleukin 10 (IL-10) [8] and transforming growth factor-beta (TGF-*β*) [9], the stimulation of inhibitory cell surface components on T cells such as programmed death-1 (PD-1) [10] and cytotoxic T-lymphocyte-associated protein 4 (CTLA-4) [11] or by activation of Fas/FasL pathway [12].

Among the distinct suppressor cell populations, MDSCs play a key role. They represent a heterogeneous population of immature myeloid cells consisting of myeloid progenitors and precursors of macrophages, granulocytes, and dendritic cells (DCs) which are characterized by their functional suppressive ability [13]. Two main subpopulations of MDSCs have been identified in humans; monocytic (M-MDSC) and granulocytic (G-MDSC) [14]. Several suppressive functions of MDSC have been suggested including inhibition of T cell (CD8^+^ and CD4^+^) activation [15], DC differentiation [16], impairment of B-cells [17], blocking natural killer (NK) cell cytotoxicity [18], and expansion of Tregs [19]. MDSCs are also involved in angiogenesis and metastasis through production of matrix metallopeptidase 9 (MMP9) and TGF-*β*1 [20]. However, the mechanisms leading to the suppression of the immune responses are highly dependent on the cancer's microenvironment [21].

In the recent years, investigation of MDSCs has spread over most of the human solid tumors. However, there is no consensus regarding the phenotypic characterization of these cells since several distinct combinations of markers have been used in different studies. Few studies have tested the expression of MDSCs in NSCLC patients and even less have identified them by using markers of immaturity of myeloid and lymphoid cells such as human leukocyte antigen-DR (HLA-DR) or Lineage according to the more recent definition of MDSCs [22]. It has been recently demonstrated that a granulocytic subtype (CD15^+^CD14^−^CD33^+^CD11b^+^) of MDSCs has suppressive activity, whereas an increased number of MDSCs (CD11b^+^CD14^−^ cells) was negatively associated with the frequency of CD8^+^ T lymphocytes and responsiveness to treatment in patients with NSCLC [23]. Moreover, it has been reported that CD33^+^CD11b^+^ MDSCs, a population with more mature features, were able to suppress T cell proliferation in NSCLC patients [24].

The aims of the current study were to identify phenotypically new subpopulations of MDSCs, strictly in immature myeloid cells, in NSCLC patients and to correlate them with the patients' clinical outcome.

## 2. Materials and Methods

### 2.1. Patients and Healthy Donors

Peripheral blood in EDTA (BD Biosciences, Europe) was obtained from 110 chemotherapy-naive NSCLC patients at the time of diagnosis and before the administration of any systemic or local treatment. All patients were older than 18 years and had not received any immunosuppressive drugs or G-CSF injections prior to immune testing. Patients' demographics are presented in Table 1. All patients were diagnosed with inoperable, locally advanced (Stage III with pleural effusion or severe respiratory failure) or metastatic (Stage IV) NSCLC. The median patients' age was 68 years, 84.5% were men, 51.8% had an adenocarcinoma, and 74.5% had stage IV disease. All patients were treated with 4–6 cycles of platinum-based chemotherapeutic regimen (13.6% and 78.3% with or without bevacizumab, resp.). Eighty-eight (86.3%) patients were evaluable for assessment of clinical outcome; the rest of them (*n* = 22) either received only one chemotherapy cycle (*n* = 14) because of early death or refused systemic anticancer treatment and received only supportive care (*n* = 8). For controls, blood samples were collected from 19 age- and sex-matched healthy (12 males and 7 females; age 68 ± 7 years) volunteers. All patients and controls provided a written informed consent and the study was approved by the ethics and scientific committees of our Institution.

### 2.2. Cell Isolation and Flow Cytometry for Immunophenotypic Analysis of Cells

Peripheral blood from chemotherapy-naive patients with advanced or metastatic NSCLC was centrifuged; the plasma was removed and was stored at −80°C. Blood samples underwent red blood cell lysis using red blood cell (RBC) lysing buffer according to the manufacturer recommendations (BD Biosciences). Briefly, 5 mL EDTA-treated whole blood was added into a tube containing 45 mL RBC lysing buffer at room temperature. Following 20 min incubation at room temperature, the tubes were centrifuged at 500 g for 5 min. The supernatant was discarded and the white blood cell pellet was washed twice with 15 mL flow buffer (1% FCS, 0.01% NaN_3_ in PBS; Sigma, USA) and cells were then resuspended in flow buffer (1 × 10^7^/mL) for immunophenotypic analysis.

**Figure 1:**
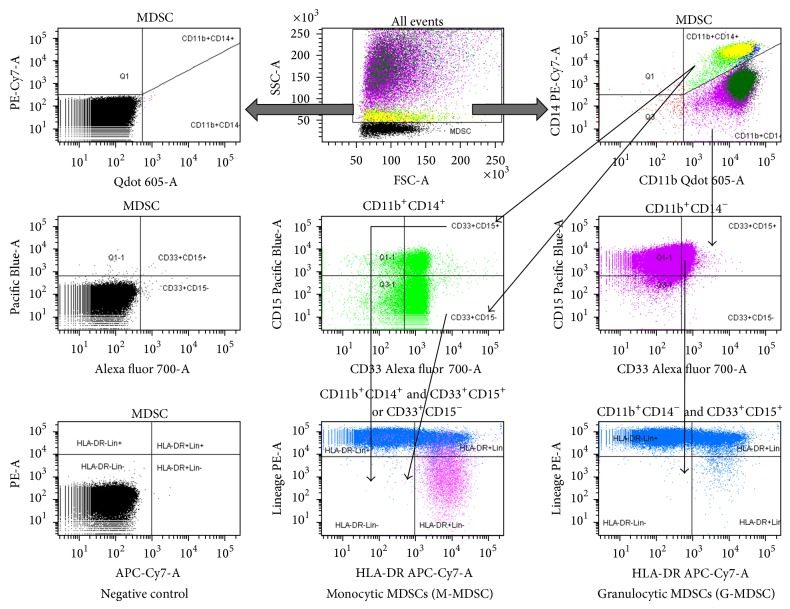
Phenotypic analysis of MDSC subpopulations in NSCLC patients. Representative dot plots, as well as the gating strategy for identification and quantification of MDSCs. Arrows indicate the sequence of gating. The gates for each dot plot and histogram are presented on the top of each box. The positive expression of markers compared to cells without Ab staining. Purple color represents the CD11b^+^CD14^−^ population whereas bright light green represents the CD11b^+^CD14^+^ population.

Fluorescence-active cell sorting (FACS) analysis was performed on freshly isolated cells. White blood cells were stained for expression of surface markers using anti-human monoclonal antibodies conjugated to fluorochrome against different molecules: (a) for the MDSCs subsets: anti-CD14 PE Cy7; anti-CD15 V450; anti-CD11b FITC; anti-CD33 Alexa 700; anti-HLA-DR APC-H7; anti-Lin (CD3/CD4/CD16/CD56/CD19) PE, (b) for B and T cells: anti-CD3 PE-CF594; anti-CD4 V500; anti-CD8 APC-Cy7, anti-CD19-FITC, and (c) for DC and monocytes: anti-CD14 PE Cy7; anti-CD11b FITC; anti-HLA-DR APC-H7. All antibodies were purchased from BD Biosciences (USA). Staining was performed for 30 min, on ice in dark. After washing, cells were resuspended in 0.5 mL flow buffer and a multicolour analysis was performed using an LSRII flow cytometer (BD Biosciences). For intracellular staining, the cells were permeabilized by BD IntraSure kit according to manufacturers' instructions and stained for inducible nitric oxide synthase (iNOS)—PerCP (Santa-Cruz, USA). Analysis of FACS data was done using FACS Diva Software (BD Biosciences). For T-cell subset and B cell analysis, the acquisition and analysis gates were restricted to the lymphocyte population, whereas for MDSC analysis, all cells, but lymphocytic mononuclear cells, were included. CD3^+^/CD4^+^ and CD3^+^/CD8^+^ cells were calculated as a percentage of CD3^+^ lymphocytes. In an attempt to be in line with the definitions of MDSCs that have been used in various tumors [22], we defined and investigated the presence of CD14 and CD15 markers strictly gated in HLA-DR and Lineage negative myeloid cells (CD33^+^CD11b^+^HLA-DR^−^Lin^−^). MDSCs were subclassified into two subsets: M-MDSC and G-MDSC. The M-MDSCs expressing HLA-DR^−^Lin^−^CD33^+^CD11b^+^CD14^−^CD15^+^ or CD15^−^ (referred to as CD14^+^CD15^+^HLA-DR^−^Lin^−^ and CD14^+^CD15^−^HLA-DR^−^Lin^−^, resp.) and the G-MDSCs expressing HLA-DR^−^Lin^−^CD33^+^CD11b^+^CD14^+^CD15^−^ (referred to as CD14^−^HLA-DR^−^Lin^−^ throughout the paper) were calculated as a percentage of CD11b^+^CD14^−^ and CD11b^+^CD14^+^, respectively. Each measurement contained 100,000 events. The gating strategy for both M-MDSCs and G-MDSCs populations is shown in Figure 1.

### 2.3. Reactive Oxygen Species (ROS) Detection

The intracellular oxidant intensity was determined by using 5-(and-6)-chloromethyl-2′,7′-dichlorodihydrofluorescein diacetate-acetyl-ester (DCFDA; Invitrogen, USA), which is metabolized to fluorescent 2′-7′-dichlorofluorescein (DCF) upon oxidation. Single-cell suspensions from blood were incubated in RPMI1640 medium containing 2.5 *μ*M DCFDA with/or without 30 ng/mL PMA for 30 min at room temperature. Subsequently, cells were washed twice in flow buffer and stained with MDSC mAbs and the mean fluorescence intensity (MFI) of intracellular DFC was determined by flow cytometry.

### 2.4. Statistical Analysis

Statistical analysis was performed using GraphPad Prism version 6.0 (GraphPad Institute Inc, USA). Data are presented as mean ± SEM. Differences between groups were determined using the Mann-Whitney nonparametric test and Wilcoxon matched-pairs signed rank test, as stated. Spearman's rank correlation tests were used to assess relationships between the levels of MDSCs and other tested immune cells types. High expression of MDSCs was defined as the percentage of the cells above the 90% percentile of the controls. Median OS and progression-free survival (PFS) were estimated using the Kaplan-Meier method with groups compared using the log-rank test. OS was defined as the time from the study enrolment to death. PFS was defined as the time between the enrolment and the first date of first observation of clinical progression or death. A univariate Cox regression analysis, with hazard ratios (HR) and 95% confidence intervals (95% CI), was used to assess the association between each potential prognostic factor with OS and PFS. All variables significant on univariate analysis were considered further in a multivariate Cox proportional hazards regression model to evaluate the independent significance of different variables on OS and PFS. Differences and associations were considered significant where *P* < 0.05.

## 3. Results

### 3.1. Phenotypic Definition of MDSCs in the Peripheral Blood in NSCLC Patients

Initially, we sought the MDSC subpopulations that have been described by other groups in NSCLC patients. Indeed, we determined the percentages of CD33^+^CD11b^+^, CD14^−^CD11b^+^, CD11b^+^CD14^−^CD15^+^, CD11b^+^CD33^+^CD14^−^CD15^+^, and CD14^+^HLA-DR^−/low^ population (Table 2(a)).

Next, we identified, phenotypically, two newly monocytic subpopulations (CD14^+^CD15^+^HLA-DR^−^Lin^−^ and CD14^+^CD15^−^HLA-DR^−^Lin^−^, resp.) and one granulocytic (CD14^−^HLA-DR^−^Lin^−^) based on the expression of CD15 and CD14 markers in the immature myeloid cell population (CD33^+^CD11b^+^HLA-DR^−^Lin^−^) in the peripheral blood of chemotherapy-naive NSCLC patients (Table 2(b)).

### 3.2. Myeloid-Derived Suppressor Cell Subsets Are Increased in Patients with NSCLC

The percentages of CD33^+^CD11b^+^ (41.4 ± 4% versus 24.4 ± 5%; *P* = 0.03), CD14^−^CD11b^+^ (66.1 ± 3% versus 48.02 ± 6%; *P* = 0.01), CD11b^+^CD14^−^CD15^+^ (83.3 ± 2.3% versus 78.4 ± 4%; *P* = 0.02), and CD11b^+^CD33^+^CD14^−^CD15^+^ (40.3 ± 3% versus 21.3 ± 5%; *P* = 0.01) cells were significantly increased in patients compared to healthy donors (Figures S1(a–d) in Supplementary Material available online at http://dx.doi.org/10.1155/2014/659294), with an exception of the CD14^+^CD11b^+^HLA-DR^−/low^ population which did not differ (25.2 ± 2% versus 24.4 ± 6%; *P* = 0.2; Figure S1(e)).

**Figure 2:**
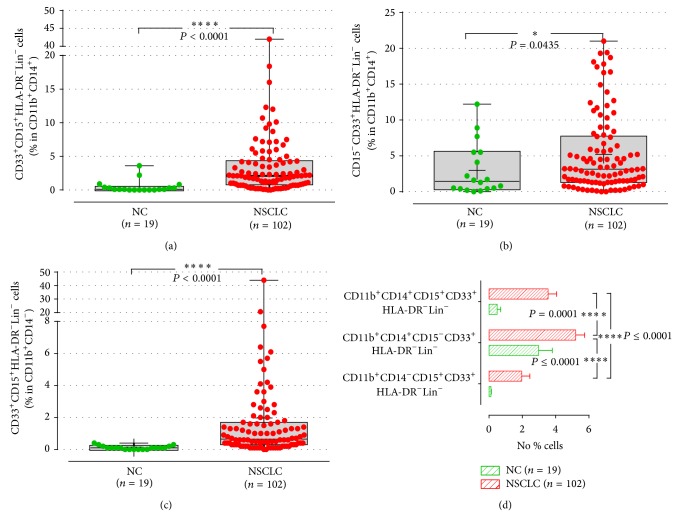
M-MDSC and G-MDSC subpopulations in NSCLC patients and normal controls. Percentage of monocytic (a), (b), and granulocytic (c) subpopulations of MDSCs in whole blood. Each point corresponds to an individual patient or healthy controls. The medians, 75 percentile (box), and max and min (whiskers) are represented.* P* values are determined by Mann-Whitney test (d). Comparison of the percentages between MDSCs subpopulations in the whole blood of the patients. Percentages indicated in the plots represent the percentages of phenotypic marker expression in the parental population, which are presented inside the brackets. The bars denote mean values ± SEM and the *P* values are determined by Wilcoxon matched-pairs signed rank test.

Elevated levels of M-MDSC subpopulations, defined as CD14^+^CD11b^+^CD33^+^CD15^+^HLA-DR^−^Lin^−^ (CD14^+^CD15^+^HLA-DR^−^Lin^−^; 3.5 ± 0.5% versus 0.5 ± 0.2%; *P* ≤ 0.0001) and CD14^+^CD11b^+^CD33^+^CD15^−^HLA-DR^−^Lin^−^ (CD14^+^CD15^−^HLA-DR^−^Lin^−^; 5.2 ± 0.5% versus 3 ± 0.8%; *P* = 0.04), were observed in patients compared to healthy donors (Figures 2(a) and 2(b)). Similarly, the levels of the G-MDSC subpopulation (CD14^−^CD11b^+^CD33^+^CD15^+^HLA-DR^−^Lin^−^; CD14^−^HLA-DR^−^Lin^−^) were significantly increased in patients (2 ± 0.5%, *n* = 102) compared to healthy controls (0.1 ± 0.02%, *P* ≤ 0.0001; Figure 2(c)).

Patients were then grouped by clinical cancer stage. The differences between normal volunteers and patients with stage III and stage IV solid tumours were also statistically significant (*P* < 0.0001). However, the differences of the MDSCs' percentages between locally advanced cancer patients (stage III) and stage IV were not significant (Figure S2).

Subsequently, we investigated whether these subpopulations are equally expanded in the peripheral blood of NSCLC patients in NSCLC favour of one subpopulation over the other. Indeed, the frequency of CD14^+^CD15^−^HLA-DR^−^Lin^−^ subpopulation was more prevalent (5.2 ± 0.5%) in the whole blood of patients compared to the other two subpopulations (CD14^+^CD15^+^HLA-DR^−^Lin^−^: 3.5 ± 0.5%, *P* ≤ 0.0001, and CD14^−^HLA-DR^−^Lin^−^: 2 ± 0.5%, *P* = 0.0001) (Figure 2(d)). However, there were significant positive correlations between each MDSC subpopulation (CD14^+^CD15^−^HLA-DR^−^Lin^−^ versus CD14^+^CD15^+^HLA-DR^−^Lin^−^, Spearman *r* ^2^ = 0.5, *P* ≤ 0.0001; CD14^+^CD15^−^HLA-DR^−^Lin^−^ versus CD14^−^HLA-DR^−^Lin^−^, Spearman *r* ^2^ = 0.2, *P* ≤ 0.05; CD14^+^CD15^+^HLA-DR^−^Lin^−^ versus CD14^−^HLA-DR^−^Lin^−^, Spearman *r* ^2^ = 0.3, *P* ≤ 0.003) indicating that all MDSCs subtypes are equally increased. In contrast, the percentages of all subtypes of MDSCs did not statistically differ in the normal control (Figure 2(d)).

### 3.3. ROS Production and iNOS Expression in Different MDSCs' Subtypes

**Figure 3:**
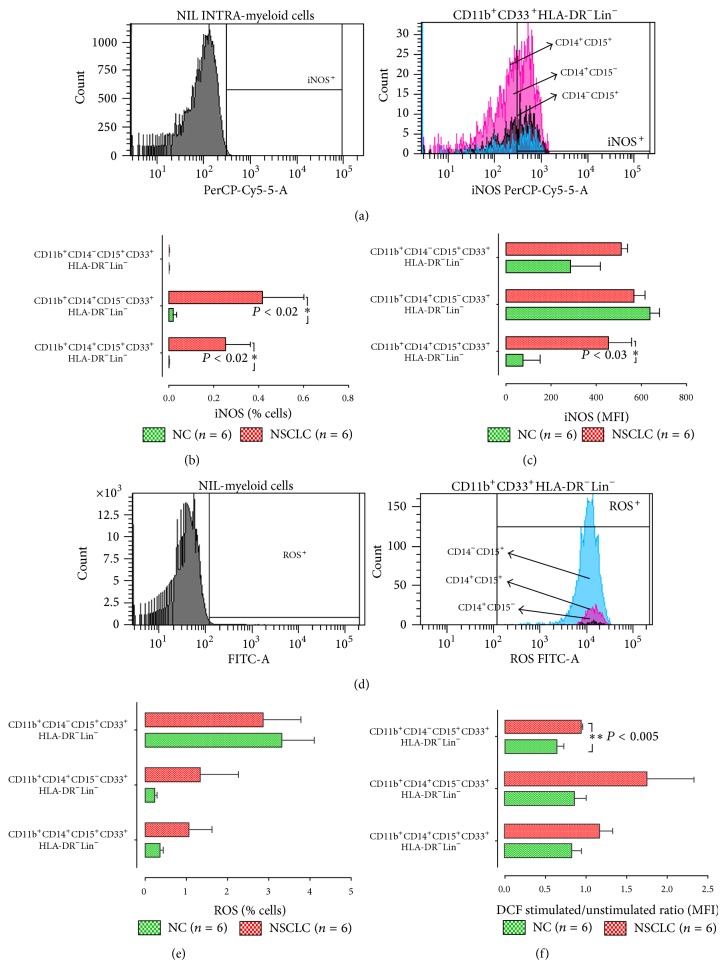
Representative histograms of flow cytometry analysis of (a) iNOS and (d) intracellular oxidative stress by the DCF method. Percentages of (b) iNOS and (e) ROS producing cells from healthy and NSCLC patients. Intracellular levels of (c) iNOS and (f) ROS in all tested subpopulations. The data are the mean fluorescence intensity (MFI). Intracellular ROS levels in subpopulations of MDSCs before and after PMA stimulation. Green bars, healthy controls; red bars, NSCLC patients. The gates for each dot plot and histogram are presented on the top of each box. The positive expression of markers is compared to cells without Ab staining. Colours in histograms represent the different subpopulations; pink colour, CD11b^+^CD14^+^CD15^+^CD33^+^HLA-DR^−^Lin^−^ population; black colour, CD11b^+^CD14^+^CD15^−^CD33^+^HLA-DR^−^Lin^−^, and bright blue, CD11b^+^CD14^−^CD15^+^CD33^+^HLA-DR^−^Lin^−^. Percentages indicated in the plots represent the percentages of phenotypic marker expression in the parental population, which are presented inside the brackets. The data are represented as the mean ± SEM and the *P* values are determined by Mann-Whitney test.

In order to investigate if these new subpopulations of NSCLC MDSCs are functional as well as their possible mechanisms of action, we assessed the expression of ROS and iNOS in all of our subpopulations by flow cytometry (Figure 3(a)). A higher frequency of both subpopulations of M-MDSCs (CD14^+^CD15^+^HLA-DR^−^Lin^−^ and CD14^+^CD15^−^HLA-DR^−^Lin^−^) expressed iNOS [0.3 ± 0.1%, (*n* = 6) and 0.4 ± 0.2%, (*n* = 6), resp., *P* < 0.02] in NSCLC patients compared to normal controls [0.002 ± 0.001%, (*n* = 6) and 0.02 ± 0.017%, (*n* = 6), resp.]. In contrast, neither NSCLC patients nor healthy controls did have iNOS-expressing CD14^−^HLA-DR^−^Lin^−^ MDSCs (Figure 3(b)). The expression levels of iNOS, as determined by mean fluorescence intensity (MFI), were also higher only in NSCLC CD14^+^CD15^+^HLA-DR^−^Lin^−^ [453 ± 99, (*n* = 6); *P* < 0.03] compared with healthy donors [75 ± 50, (*n* = 6); Figure 3(c)].

Next, we assessed ROS levels in M-MDSCs and G-MDSCs subpopulations by measurement of 2′-7′-dichlorofluorescein (DCF) (Figure 3(d)). Even though the percentage of G-MDSCs (CD14^−^HLA-DR^−^Lin^−^-) producing ROS was numerically higher in patients compared to other subpopulations and healthy controls, this difference could not reach statistical significance (Figure 3(e)). However, in patients, the levels of ROS production by G-MDSCs following PMA stimulation was significantly higher [1 ± 0.03, (*n* = 6), *P* < 0.005] than the healthy controls' G-MDSCs [0.6 ± 0.08, (*n* = 6); Figure 3(f)].

### 3.4. Percentages of CD4^+^ T Cells, Dendritic Cells/Monocytes in Treatment-Naive NSCLC Patients

Phenotypic analysis of other immune cells demonstrated a decrease in the percentages of CD3^+^CD4^+^ T helper cells (36 ± 2.1% versus 58.7 ± 4.5%, *P* ≤ 0.0001), CD14^+^CD11b^+^ monocytes (18.8 ± 1.7% versus 34.4 ± 4.6%, *P* ≤ 0.0004), and mature CD14^+^HLA-DR^+^ DC (47.6 ± 2.6% versus 63.1 ± 5.2, *P* = 0.015) in NSCLC patients compared to normal controls. On the contrary, there was no difference in the expression of CD19^+^ B cells (8.2 ± 0.8% versus 20.1 ± 2%, *P* = 0.6) and CD3^+^CD8^+^ cytotoxic T cells (20.6 ± 1.2% versus 7.4 ± 0.9%, *P* = 0.9; Table 3).

### 3.5. Relationship between MDSC Subpopulations and Other Immune Cells

Further analysis revealed that the percentage of both subpopulations of M-MDSCs, but not of G-MDSCs subpopulation, was inversely correlated with DC/monocytes percentages (CD14^+^CD15^+^HLA-DR^−^Lin^−^ versus DC/monocytes: Spearman *r* ^2^ = −0.3, *P* ≤ 0.001; CD14^+^CD15^−^HLA-DR-Lin^−^ versus DC/monocytes: *r* ^2^ = −0.201, *P* ≤ 0.04). Moreover, the G-MDSCs subpopulation, but not the M-MDSCs subpopulations, was inversely correlated with the levels of CD4^+^ T cells (CD14^−^HLA-DR-Lin^−^ versus CD4^+^ T: *r* ^2^ = −0.3, *P* = 0.006).

### 3.6. Response to Treatment according to Baseline Immunological Parameters

**Figure 4:**
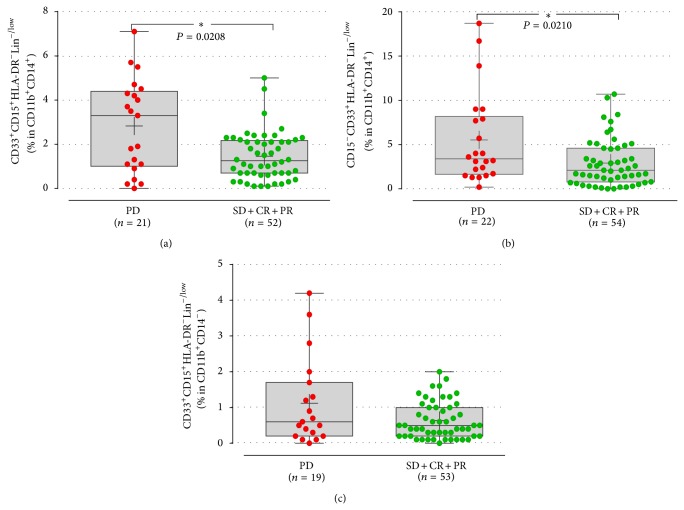
Response to 1st line treatment in patients according to the MDSC expression at baseline. The percentages of both monocytic (a) and (b), but not the granulocytic (c), subpopulations of MDSCs were increased in patients with disease progression (PD) compared to those with disease control after therapy. Each point corresponds to an individual patient or healthy controls. The medians, 75 percentile (box), and max and min (whiskers) are represented. Groups were compared by Mann-Whitney test.

Patients with progressive disease (PD) upon front-line chemotherapy had significantly increased percentages of both M-MDSC subpopulations of (CD14^+^CD15^+^HLA-DR^−^Lin^−^: 1.1 ± 0.3%; CD14^+^CD15^−^HLA-DR^−^Lin^−^: 5.5 ± 1.1%) compared to those with disease control (DiC) (0.6 ± 0.07%, *P* = 0.02; 2.9 ± 0.3%; *P* = 0.02, resp.; Figures 4(a) and 4(b)). In contrast, the G-MDSCs did not correlate with the response to treatment (PD versus DiC: 1.1 ± 0.2% versus 0.6 ± 0.07%, *P* = 0.3, resp.; Figure 4(c)).

**Figure 5:**
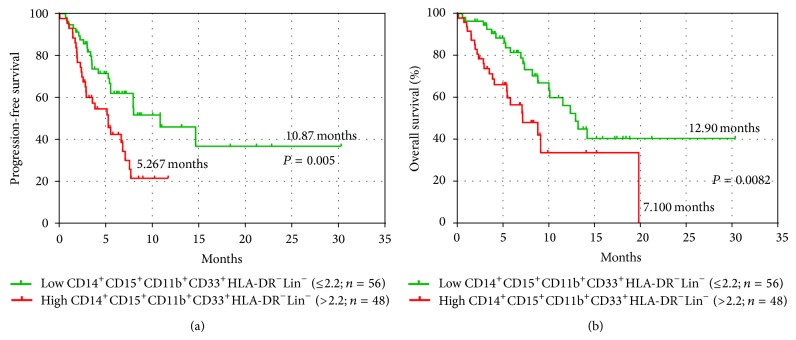
Kaplan-Meier plots of OS and PFS in patients according to the percentages of monocytic subpopulation (CD14^+^CD15^+^CD11b^+^CD33^+^HLA-DR^−^Lin^−^) of MDSCs before any systemic treatment. Comparison of (a) progression-free survival (PFS) and (b) overall survival (OS) between normal (≤2.2%) and increased (>2.2%) percentages of CD14^+^CD15^+^CD11b^+^CD33^+^HLA-DR^−^Lin^−^MDSC.

Assuming increased levels of MDSCs those that were over the 90% percentile of the controls (outliers excluded) patients were dichotomized into those with above normal range of MDSC percentage (high expression > 2.2%) and those within the normal range (≤2.2%). The detection of CD14^+^CD15^+^HLA-DR^−^Lin^−^ MDSCs within the normal levels at baseline was associated with longer PFS and OS compared to those with high levels (10.87 versus 5.3 months *P* = 0.005 and 12.9 versus 7.1 months, *P* = 0.008, resp.; Figure 5). On the other hand, neither the levels of CD14^+^CD15^−^HLA-DR^−^Lin^−^ nor CD14^−^HLA-DR^−^Lin^−^ subpopulation correlated with the clinical outcome of the patients.

### 3.7. Correlation of M-MDSC Levels with the Patients' Clinical Outcome

Univariate analysis revealed that high expression of CD14^+^CD15^+^HLA-DR^−^Lin^−^ M-MDSCs (*P* = 0.003) and disease stage (*P* = 0.03) significantly correlated with decreased PFS whereas only high expression of CD14^+^CD15^+^HLA-DR^−^Lin^−^ M-MDSCs was significantly associated with decreased OS (Table 4(a)). The multivariate analysis showed that high levels of CD14^+^CD15^+^HLA-DR^−^Lin^−^ M-MDSCs emerged as an independent prognostic factor for decreased PFS (HR = 2.41; 95% CI, 1.37–4.24, *P* = 0.002) and OS (HR = 2.35; 95% CI, 1.25–4.41, *P* = 0.008; Table 4(b)).

## 4. Discussion

Nowadays, it is widely accepted that there are distinct tumor-mediated mechanisms observed in cancer patients and impede the adequate immune response against tumor cells. Among them, MDSCs play a crucial role and their importance is a subject of extensive investigation. Phenotypic and functional heterogeneity of these cells creates many difficulties in the identification of MDSCs in humans. Studies in cancer patients with different tumor types suggest various MDSCs definitions based, mainly, on the expression of CD33, CD11b, and CD15 molecules and by the absence or low levels of the HLA-DR molecule [22]. Two major subtypes of MDSCs have been described in humans, the M-MDSCs that express predominantly the CD14 molecule and the G-MDSCs that express CD15 molecule. In prostate cancer [25] and melanoma patients [26], increased levels of CD14^+^CD11b^+^HLA-DR^low/−^ M-MDSCs have been associated with poor immune response to an antitumor vaccine. In addition, high percentages of CD14^+^HLA-DR^−/low^ M-MDSCs in patients with hepatocellular carcinoma were shown to induce the production of CD4^+^CD25^+^Foxp3^+^ regulatory T cells [19]. On the other hand, in patients with renal cell carcinoma, an increased expression of a G-MDSC subtype was detected [27], whereas other studies in colon carcinoma and melanoma have shown increased levels of both G-MDSC and M-MDSC subtypes [28]. Recent reports in NSCLC have defined MDSCs as CD33^+^CD11b^+^ [24], CD14^−^CD11b^+^ [23], CD15^+^CD14^−^CD33^+^CD11b^+^ [23], CD14^+^HLA-DR^−/low^ [29], and CD15^−^CD14^+^CD33^+^CD11b^+^ [30] cells.

In the current study, we investigated all the above mentioned subpopulations of MDSCs in patients with NSCLC and we confirmed that many of them were significantly increased compared to the healthy control (Figures S1(a–d)). Interestingly, it is demonstrated, for the first time, the presence of two monocytic CD14^+^CD11b^+^CD33^+^CD15^+^HLA-DR^−^Lin^−^ and CD14^+^CD11b^+^CD33^+^CD15^−^HLA-DR^−^Lin^−^ and a granulocytic CD14^−^CD11b^+^CD33^+^CD15^+^HLA-DR^−^Lin^−^ subpopulation of MDSCs associated with the clinical outcome in NSCLC patients (Table 4), although there was no difference in the frequency of the distinct subpopulations of MDSCs according to the histology, stage, and subsequent chemotherapy treatment (data not shown). The M-MDSC subpopulations were the predominant subtypes, as they were markedly increased compared to G-MDSC in contrast with the data coming from preclinical studies which reported that the G-MDSC subtype was predominant in tumor-bearing mice [31]. However, Movahedi et al. showed that both subtypes were equally increased in mice bearing T cell lymphoma [32]. This observation seems to indicate that regulation of MDSCs differentiation can be tumor-driven by taking into consideration that different tumor-factors are released in different cancer types [32]. Therefore, the significance of the increased levels of M-MDSCs over the granulocytic subtype in NSCLC needs to be further investigated by a direct comparison of their expression in distinct cancers.

Several studies have suggested a negative impact of MDSC in the effector cells of the immune system of cancer patients, including NK cells [33], DCs, both lymphocytic populations (CD4^+^ and CD8^+^ T cells), B cells, and monocytes [34, 35]. Indeed, in the present study a dramatic reduction in the numbers of mature DC (CD14^+^HLA-DR^+^), the key regulators in the activation of lymphocyte subsets to control or eliminate tumors, was observed. In addition, the levels of monocytes (CD14^+^CD11b^+^), as well as of CD3^+^CD4^+^ T cells were also found significantly decreased compared to healthy controls. On the contrary, the levels of CD3^+^CD8^+^ cytotoxic T cells, as well as CD19^+^ B cells did not differ between the NSCLC patients and controls. Moreover, the reduced levels of the effector cells were inversely correlated with the presence of the MDSCs suggesting that the circulating MDSCs exert a negative regulation on the effector immune cells. Whether this negative regulation could be attributed to direct contact of MDSCs and the effector cells or other underlying indirect mechanisms such as release of cytokines is a subject of extensive investigation.

Identification of specific prognostic/predictive markers in NSCLC is of high importance in order to facilitate the selection of patients who most likely will respond to the treatment or even those who might need an immune intervention. Only few studies, so far, have provided evidence for the prognostic and/or predictive value of the elevated levels of MDSC in cancer patients. Brimnes et al. have shown increased expression of CD14^+^HLA-DR^−/low^ M-MDSC in the blood of multiple myeloma patients at the time of diagnosis compared to patients with disease remission [36]. Similarly, increased levels of CD11b^+^CD33^+^HLA-DR^−^Lin^−/low^ MDSC were shown as an independent prognostic factor in pancreatic, oesophageal, and gastric cancers and were associated with an increased risk of death [37]. Recently, two clinical studies have shown that high levels of CD14^+^HLA-DR^−/low^ and CD11b^+^CD14^−^CD15^+^ MDSCs were negatively associated with survival in renal cell carcinoma patients [38]. In NSCLC patients few studies have correlated the high MDSC levels with the clinical outcome and none of them has reported a correlation of MDSC levels with the OS. Liu et al. [23] demonstrated that a G-MDSC subtype (CD15^+^CD14^−^CD33^+^CD11b^+^) was negatively associated with responsiveness to treatment. However, in our study, the G-MDCS subpopulation did not correlate with the clinical outcome of the patients which might be interpreted by the fact that this subpopulation in Liu's study had been defined in PBMCs after Ficoll isolation. Two other studies have correlated the increased levels of circulating CD14^+^HLA-DR^−/low^ and CD11b^+^CD14^+^S100A9^+^ cells with worse PFS and response to treatment [29, 30], but none with the OS. Moreover, all of the studies did not defined MDSCs as strictly immature myeloid cells defined by the complete lack of the HLA-DR and lineage molecules.

An interesting finding of the present study is that baseline M-MDSCs levels had a clear predictive denotation since disease progression during or after chemotherapy was correlated to elevated values of these cells. In our study, it is emerged for the first time the independent predictive and prognostic value of CD14^+^CD15^+^HLA-DR^−^Lin^−^ but not the CD14^+^CD15^−^HLA-DR^−^Lin^−^ monocytic MDCSs, as revealed by multivariate analysis. Normal levels of CD14^+^CD15^+^HLA-DR^−^Lin^−^ MDSCs at baseline were associated with a better patients' PFS and OS compared to patients with high levels.

A limitation of the current study is the absence of functional data demonstrating the suppressive activity of the newly identified subpopulations of MDSCs. This was beyond the scope of the study which was the clarification of new, more specific, subpopulations of MDSCs and interestingly, their possible correlation with the patients' clinical outcome. Several studies have clearly shown that the immunosuppressive effect of the MDSCs is mediated by distinct mechanisms. The function of MDSC is highly depended on the cancer microenvironment and mediates its suppressive activity on the immune system through multiple mechanisms such as the production of ROS, nitric oxide (NO), and arginase (ARG-1) and secretion of the cytokines IL-10 and TGF-*β*1 [21]. The activity of ROS with NO forms peroxynitrite affects the conformational flexibility of TCR and its interaction with peptide-MHC complex [39]. On the other hand, ARG-1 contributes to the downregulation of the CD3*ζ* chain expression of the T cell receptor causing T cell arrest [27]. In the present study, we demonstrated that G-MDSC subpopulation exerts its function by producing ROS, while M-MDSCs work rather through expression of iNOS (Figure 3). Apparently, further functional studies are required for determination of specific immune suppressive activity of these novel MDSC subpopulations.

To conclude, in the present study, for first time, three distinct subpopulations of MDSCs were identified in NSCLC patients, two of monocytic and one of granulocytic origin. These subsets were independently associated with the patients' clinical outcome and thus they could be considered as potential, predictive, and/or prognostic factors. The observation that their increased expression was correlated with reduced percentages of effector cells, such as dendritic and T-helper cells could lead to the hypothesis that their elimination would likely restore the host's antitumor immune activity and consequently would improve the clinical outcome.

## Supplementary Material

Supplementary figure 1 (S1) provides individual patient's percentages of immunosuppressive MDSCs subpopulations in the peripheral blood of NSCLC patients before any systemic treatment compared to healthy donors (p value is determined by Mann-Whitney test).
In the supplementary figure 2 (S2), percentages of monocytic-MDSC and granulocytic-MDSC subpopulations in NSCLC patients according to tumour stage are showed before any treatment (p values are determined by Mann-Whitney test).

## Figures and Tables

**Table 1:** Patients' demographics.

	Patients (*n* = 110)	%
Median age		
Years (range)	68 (53–89)	
Sex		
Male	93	84.5
Female	17	15.5
Histology		
Adenocarcinoma	57	51.8
Squamous	37	33.6
Other types	16	14.6
Stage		
IIIA/B (noneligible for radiation)	28	25.5
IV	82	74.5
Treatment regimens		
Platinum-based	86	78.3
Taxane-based + bevacizumab	15	13.6
Taxane (single agent)	5	4.5
Taxane (single agnet) + bevacizumab	4	3.6
Response to therapy		
PR	25	22.7
SD	39	35.5
PD	24	21.8
NE	22	20.0

NE: nonevaluated, PR: partial response, SD: stable disease, PD: progressive disease.

**Table:** (a) Published subpopulations

MDSC subpopulations (%) (parental gate)	NSCLC patients (*n* = 110)		
	Mean ± SEM	Range	Median
CD33^+^ CD11b^+^ (in CD11b^+^)	41.41 ± 2.9	0.2–97.9	40.80
G-MDSC			
CD14^−^CD11b^+^ (lymphocytes excluded)	66.07 ± 3.0	0.7–99.9	74.70
CD14^−^CD11b^+^CD33^+^ (in CD14^−^CD11b^+^)	83.25 ± 2.3	3.6–99.9	93.65
CD14^−^CD15^+^CD33^+^CD11b^+^ (in CD14^−^CD15^+^)	40.27 ± 3.1	0.1–97.3	40.40
M-MDSC			
CD14^+^HLA-DR^−/low^ (in CD14^+^)	25.2 ± 2.1	0.8–83.2	19.01

**Table:** (b) New subpopulations

MDSC subpopulations (%) (parental gate)	NSCLC patients (*n* = 110)		
	Mean ± SEM	Range	Median
G-MDSC			
CD14^−^CD15^+^CD33^+^CD11b^+^HLA-DR^−^Lin^−^ (in CD14^−^CD15^+^)	1.97 ± 0.5	0–44	0.65
M-MDSC			
CD14^+^CD15^+^CD33^+^CD11b^+^HLA-DR^−^Lin^−^ (in CD14^+^CD15^+^)	3.55 ± 0.5	0–42	2.10
CD14^+^CD15^−^CD33^+^CD11b^+^HLA-DR^−^Lin^−^ (in CD14^+^CD15^−^)	5.21 ± 0.5	0–21	3.15

M-MDSC: Monocytic Myeloid-Derived Suppressor cells; G-MDSC: Granulocytic Myeloid-Derived Suppressor cells.

**Table 3:** T cells, B cells, DC/monocytes in the blood of NSCLC patients and healthy controls. Percentages of the cells in the peripheral blood of NSCLC patients and healthy controls as obtained by flow cytometry analysis. Percentages indicated in the plots represent the percentages of phenotypic marker expression in the parental population, which are presented in brackets. Data presented as Mean ± SEM of the. (^∗,∗∗∗,∗∗∗∗^ *P* < 0.05, 0.001, 0.0001, resp.).

Cells	Healthy donors (*n* = 19)	NSCLC patients (*n* = 110)
	% cells ± SEM	% cells ± SEM
CD3^+^CD4^+^ (in lymphocytes)	58.7 ± 4.5	36.4 ± 2.1^****^
CD3^+^CD8^+^ (in lymphocytes)	20.1 ± 2	20.6 ± 1.2
CD19^+^ (in lymphocytes)	7.40 ± 0.9	8.20 ± 0.8
CD11b^+^CD14^+^ (lymphocytes excluded)	34.4 ± 4.6	18.8 ± 1.7^***^
CD14^+^HLA-DR^+^Lin^−^ (CD14^+^ cells)	63.1 ± 5.2	47.6 ± 2.6^*^

**Table:** (a) Univariate analysis

	Hazard ratio (95% CI)	*P* value
PFS		
Age (≥70 vs <70)	1.628 (0.958–2.765)	0.072
Gender (male vs female)	1.817 (0.723–4.566)	0.204
Histology (Non-Adeno vs Adeno)	1.075 (0.632–1.828)	0.789
Stage (IV vs IIIA/IIIB)	**2.150 (1.078–4.288)**	**0.030**
CD14^+^CD11b^+^CD33^+^CD15^+^HLA-DR^−^Lin^−^ (above vs below 90% of controls)	**2.343 (1.331–4.124)**	**0.02**
CD14^+^CD11b^+^CD33^+^CD15^−^HLA-DR^−^Lin^−^ (above vs below 90% of controls)	1.076 (0.458–2.526)	0.866
CD14^−^CD11b^+^CD33^+^CD15^+^HLA-DR^−^Lin^−^ (above vs below 90% of controls)	1.115 (0.603–2.063)	0.729
OS		
Age (≥70 vs <70)	1.516 (0.849–2.708)	0.160
Gender (male vs female)	2.553 (0.792–8.233)	0.117
Histology (Non-Adeno vs Adeno)	1.006 (0.562–1.800)	0.984
Stage (IV vs IIIA/IIIB)	2.060 (0.947–4.481)	0.068
CD14^+^CD11b^+^CD33^+^CD15^+^HLA-DR^−^Lin^−^ (above vs below 90% of controls)	**2.349 (1.252–4.407)**	**0.008**
CD14^+^CD11b^+^CD33^+^CD15^−^HLA-DR^−^Lin^−^ (above vs below 90% of controls)	1.697 (0.521–5.527)	0.344
CD14^−^CD11b^+^CD33^+^CD15^+^HLA-DR^−^Lin^−^ (above vs below 90% of controls)	1.071 (0.556–2.063)	0.836

**Table:** (b) Multivariate analysis

	Hazard ratio (95% CI)	*P* value
PFS		
Stage (IV vs IIIA/IIIB)	1.838 (0.918–3.680)	0.086
CD14^+^CD11b^+^CD33^+^CD15^+^HLA-DR^−^Lin^−^ (above vs below 90% of controls)	**2.408 (1.368–4.241)**	**0.002**
OS		
CD14^+^CD11b^+^CD33^+^CD15^+^HLA-DR^−^Lin^−^ (above vs below 90% of controls)	**2.349 (1.252–4.407)**	**0.008**

## Abbreviations

MDSCs: Myeloid-derived suppressor cells
DC: Dendritic cells
OS: Overall survival
PFS: Progression-free survival
PD: Progressive disease
M-MDSC: Monocytic-MDSCs
G-MDSC: Granulocytic-MDSCs.
