# Supplementary material for: A Circulating Subpopulation of Monocytic Myeloid-Derived Suppressor Cells as an Independent Prognostic/Predictive Factor in Untreated Non-Small Lung Cancer Patients
Source: J Immunol Res. 2014 Nov 11;2014:659294. doi: 10.1155/2014/659294 (PMC4243712; doi:10.1155/2014/659294)
Supplement: Supplementary file 1 — Supplementary figure 1 (S1) provides individual patient's percentages of immunosuppressive MDSCs subpopulations in the peripheral blood of NSCLC patients before any systemic treatment compared to healthy donors (p value is determined by Mann-Whitney test). In the supplementary figure 2 (S2), percentages of monocytic-MDSC and granulocytic-MDSC subpopulations in NSCLC patients according to tumour stage are showed before any treatment (p values are determined by Mann-Whitney test). [file 659294.f1.pdf]

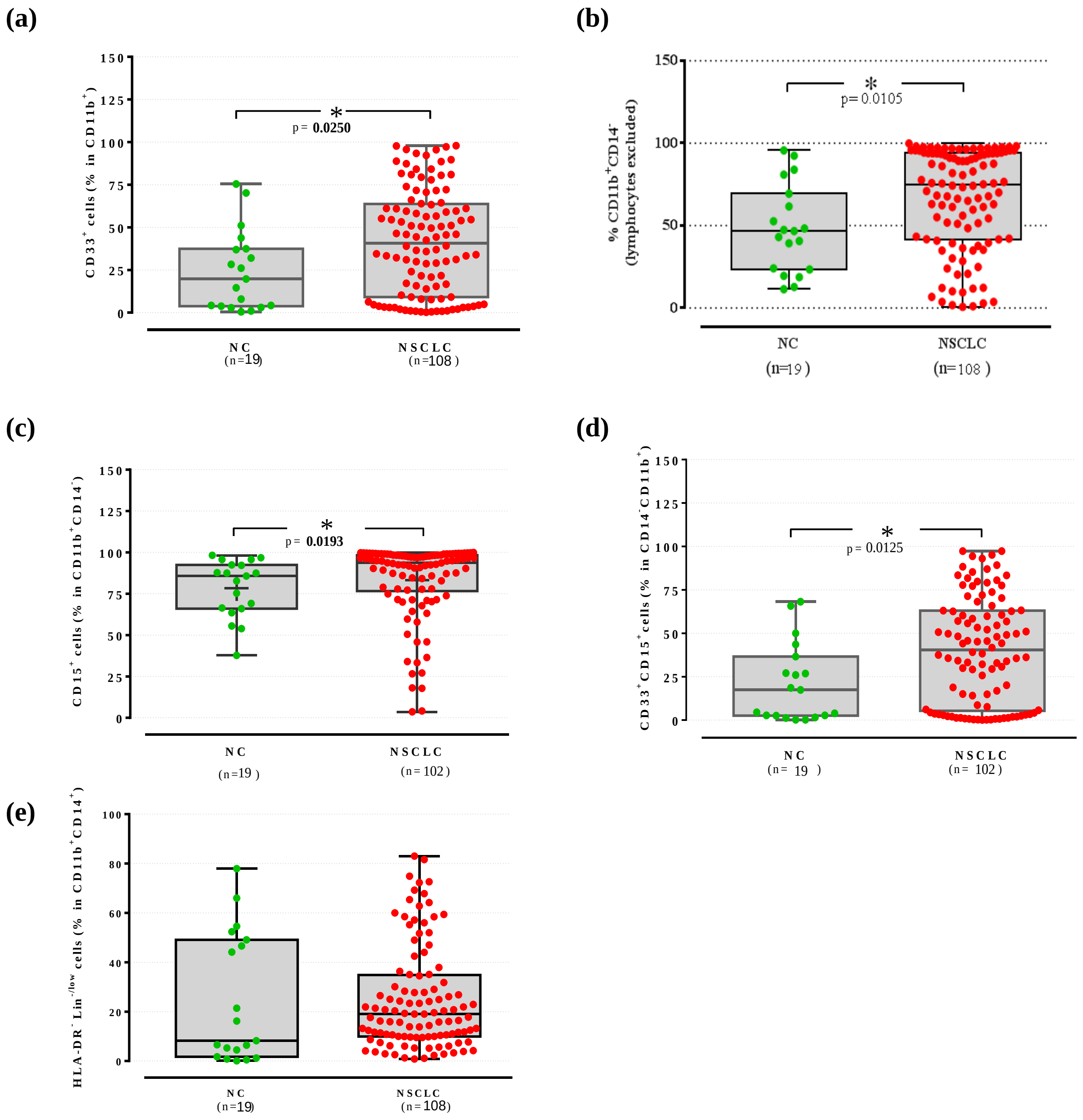

FIGURE S1. Immunosuppressive MDSCs in the peripheral blood of NSCLC patients before any systemic treatment. (A) CD33^{+}CD11b^{+}, (B) CD14^{-}CD11b^{+} (C) CD11b^{+}CD14^{-}CD15^{+} and (D) CD11b^{+}CD33^{+}CD14^{-}CD15^{+} and (E) CD14^{+}CD11b^{+}HLA-DR$^{-/low}$. Each point corresponds to an individual patient or healthy controls. The medians, 75 percentile (box) and max and min (whiskers) are represented. Percentages indicated in the plots represent the percentages of phenotypic marker expression in the parental population, which are presented inside the brackets. Groups were compared by Mann-Whitney test.

(a)

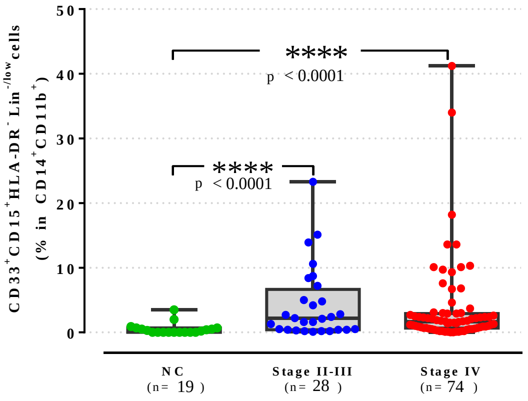

(b)

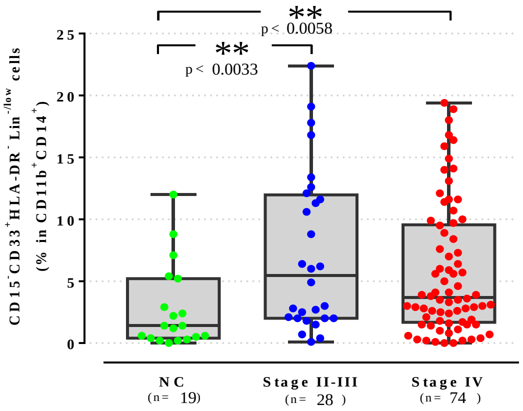

(c)

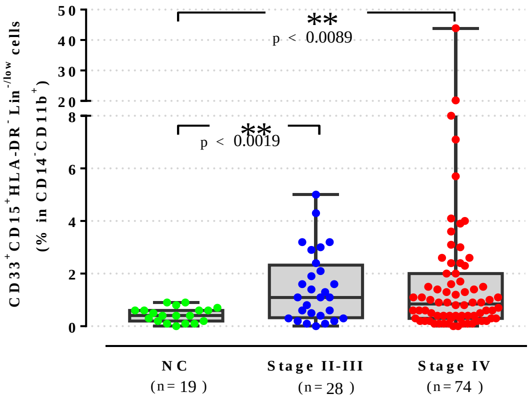

FIGURE S2. Percentages of M-MDSC and G-MDSC subpopulations in NSCLC patients do not correlate with tumour stage. Percentages of monocytic (a, b) and granulocytic (c) subpopulations of MDSCs in whole blood of NSCLC patients and healthy controls. Differences between healthy controls and NSCLC patients of all stages were statistically significant. In contrast, differences in percent of MDSCs subpopulations between stage IV cancer patients and stages III were not statistically significant. Each point corresponds to an individual patient or healthy controls. The medians, 75 percentile (box) and max and min (whiskers) are represented. P values are determined by Mann-Whitney test.
